# A Novel Noncooperative Behavior Management Method for Multiattribute Large Group Decision-Making

**DOI:** 10.1155/2022/6978771

**Published:** 2022-02-14

**Authors:** Xiaoqin Dong, Ying Yang, Bo Shao, Xianbin Sun

**Affiliations:** ^1^College of Hydraulic & Environmental Engineering, China Three Gorges University, Yichang 443002, China; ^2^School of Civil Architecture and Environment, Hubei University of Technology, Wuhan 430068, China

## Abstract

In multiattribute large-group decision-making (MALGDM), the ideal state indicates a high degree of consensus for decision-makers. However, it is difficult to reach a consensus because the conflict between various decision attributes and decision-makers increases. To deal with the problem, a novel consensus model was developed to manage the decision-making in large groups based on noncooperative behavior. The improved clustering method was used to take account of the similarities among different decision-makers, while similar decision-makers will be grouped into the same group. Moreover, the consensus threshold was determined from an objective and subjective aspect to judge whether the consensus reaching process continues. The noncooperative behavior and adjustment amount of decision-makers' opinions were investigated based on the proposed consensus model, and an emergency decision-making problem in flood disaster is applied to manifest the feasibility and distinctive features of the proposed method. The results show the proposed novel consensus model demonstrated strong applicability and reliability to the noncooperative subgroup problem and can be explored to manage multiattribute interactions in LGDM.

## 1. Introduction

Decision-making, which aims at identifying an ideal alternative based on the information described by decision-makers, is widely used in all aspects of modern life [1–3]. With the increase in the complexity of decision-making problems, many attributes relevant to decision-making problems have been explored [4]. Decision-makers need to consider all relevant aspects of the problem [5]. The decision-making behavior of decision-makers depends on many factors, including their personal and professional goals, interests, and the experience they pursue to develop themselves professionally [6]. Decision-makers need to know about Business, Management and Accounting, Engineering, Social Sciences, and Computer Science to inform the decision-making process [7]. Group decision-making (GDM) has attracted increasing attention due to its characteristic superiority of gathering knowledge of decision-makers from various fields [8–10]. Problems always involve many interconnected fields, and the decision-making results are related to the benefits of stakeholders. Thus, it becomes uneasy for small-group decision-making to reach the demands of social development [11, 12]. As the number of people in the decision-making group increases, the problem of multiattribute large group decision-making appears [13].

Clustering processing, the essential part of large group decision-making, is a fundamental but indispensable process in multiattribute large-group decision-making (MALGDM) [14]. The clustering process in MALGDM can improve the efficiency of the decision process [15]. The C-means algorithm [16] and the k-means algorithm [17] are the most available clustering methods in applications. Xue used the Choquet integral (CI) operator to measure each attribute and then aggregated it [18]. Based on Shannon entropy, Li measured the uncertainty of discrete Z-numbers by a new technique [19]. The main features of these algorithms are the early decided clustering numbers and the effect of threshold selection on classification results. Having defined the shape similarity measure, Tapia-Rosero et al. developed a clustering method, in which similar-shaped membership functions are grouped by agglomerative hierarchical clustering technique [20]. However, this method did not consider the similarity of decision-makers in the same cluster, and there exist errors for the shape similarity measure [21]. Besides, a clustering method based on vector space was proposed by Xu et al. This method considered the similarity of decision-makers' decisions [22, 23]. However, these methods classified the cluster by calculating the similarities between the decision-makers and the cluster. But those methods did not consider the similarities between two decision-makers in the same clusters. The similarities between decision-makers will decrease as the clustering processes develop.

The consensus level is selected to describe decision-makers' differences in opinion and preference. It can also measure the degree of agreement among different clusters in MALGDM [24]. There are many approaches to compute the consensus level [25–27]. In MALGDM, before making a group decision, a consensus reaching process is usually applied to reach the collective agreement [28, 29]. To reach a consensus, some decision-makers must modify their opinions in the dynamic and iterative group discussions [30]. For reaching a consensus process in MALGDM, many scholars do a lot of studies. Pérez et al. introduced a feedback mechanism and built a consensus model based on decision-makers' relevance or importance level [31]. For MALGDM problems, Xu et al. promoted the consensus reaching process in two stages. The novel method could appropriately adjust the preference of decision-makers and the weight of subgroups [32]. Jin et al. adopted a local adjustment strategy to retain preference evaluation of decision-makers as far as possible in group decision-making [33]. To reach consensus, Xu et al. proposed a dynamic consensus method based on an exit authorization mechanism. They thought the subgroup would be suggested to exit the decision-making process once the proximity index could not meet the requirement, and then the delegation mechanism is employed to reserve the cluster's influence by giving trust weights to other clusters [24]. Noncooperative behavior is common in the practical process of decision-making. However, these studies are lacking at considering the influence of noncooperative behavior. To reach a consensus, a suitable method should be adopted to manage the noncooperative behavior. Based on a self-management mechanism of noncooperative behaviors, Dong et al. introduced three kinds of noncooperative behaviors and developed a new consensus framework [34]. They also proposed a novel framework based on a self-management mechanism for noncooperative behaviors in large-scale consensus reaching processes [35]. Palomares provided a consensus model suitable to handle and detect the noncooperative behavior, and the consensus could be reached by decreasing the weight of noncooperative cluster [36]. Wu and Xu proposed a consensus model, in which the clusters can be changed. The clusters can modify if the individuals are able to change their preferences via the consensus reaching process [37]. Quesada et al. introduced a methodology to process noncooperative behaviors, in which a uniform-based weighting scheme was adopted to compute the weight of decision-makers in LGDM [38]. Nazari et al. used dynamic noncooperative games to model these conflicts when stakeholders appear noncooperative behavior [39]. With the purpose of managing minority opinion and noncooperative behavior in MALGDM, Xu and put forward the concept of comprehensive adjustment coefficient and designed an improved consensus model [40].

To sum up, problems have evolved into many interrelated areas, and the decision-making of large groups needed to meet the requirements of social development. But regarding LGDM, it is not easy to reach the consensus level in decision processes. The goal of large group decision-making is to find a method that can reach consensus effectively in large groups, improve the consensus level in short time, and obtain the accurate decision-making result. In the previous literature, the subgroup obtained in these studies remains unchanged in the consensus reaching process. This is often untrue because decision-makers modify their available opinion. The subgroup opinion must be changed when decision-makers modify their opinion. This will lead to unacceptable results of large group decision making. Meanwhile, the decision-makers in the noncooperative subgroup who are willing to modify their decision opinion should not be penalized. Unlike previous literature, this paper protected individual decision opinion. And previous methods did not consider the similarity of decision-makers in the same cluster. This will reduce the consistency of the group. Mandal et al. acknowledged noncooperative behaviors and divided them with the experts' similar evaluations into a subgroup. But decision-makers were still unable to change subgroup [41]. Therefore, it is necessary to seek a large group decision-making method that can protect the opinions of decision-makers and improve the degree of consensus. The main contribution of the paper is a precise consensus reaching model that we proposed to manage noncooperative behaviors in MALGDM. And an improved clustering method is developed. This method considers the similarities among decision-makers.

The remaining part of the paper is structured as follows: firstly, decision-makers are clustered, and the group consensus level is obtained in Section 2. In Section 3, the noncooperative behavior is detected and managed. A typical example, applied to indicate the utility and applicability of this model, is shown in Section 4. Then, Section 5 discusses the advantages and innovations of the proposed methods in detail. Finally, conclusion and future researches are provided in Section 6.

## 2. Preliminaries

### 2.1. Problem Formulation

MALGDM problems can be defined as a situation where a large number of decision-makers must make a high-quality decision result by choosing the *n* alternatives. The main parameters of MALGDM are as follows:

1. A discrete finite set of alternatives *x*={*x*_1_, *x*_2_, ⋯*x*_*n*_}, where *x*_*i*_ represents alternative solution.
2. A set of decision-makers can be denoted as *E*={*e*_1_, *e*_2_ … *e*_*M*_}(*M* ≥ 2). The weight vector of decision-makers is *ω*={*ω*_1_, *ω*_2_, ⋯*ω*_*M*_}, where  *ω*_*j*_(*j*=1,2 … *M*) ∈ (0,1) and ∑_*j*=1_^*M*^*ω*_*j*_=1. After collecting a lot of previous literature, we can determine the range of the number of large groups of decision makers. Usually, when the number of experts in a group reaches 20, that is, *M* ≥ 20, the group is considered as a large group. And the decision-making process in which they participate can be defined as large group decision-making [42, 43].
3. A finite set of attributes *F*={*f*_1_, *f*_2_ … *f*_*P*_}(*P* ≥ 2), the weight vector of attribute *η*={*η*_1_, *η*_2_, ⋯*η*_*P*_}, where *η*_*k*_ ≥ 0(*k*=1,2,…*P*), and ∑_*k*=1_^*P*^*η*_*k*_=1.

Each decision-maker will give a numerical decision matrix to express the opinion for the alternatives *A*^*j*^=(*a*_*ik*_^*j*^)_*n*×*P*_, *j* ∈ *M*, where *a*_*ik*_^*j*^ represents decision-maker *e*_*j*_'s preference of alternative *x*_*i*_ concerning attribute *f*_*k*_. The premise of this paper is that there is disagreement among decision-makers in the group. So, it is not possible for all decision-makers to behave the same preference for *x*_*i*_. This represents that $\underset{i}{\text{max}}\left\{a_{ik}^{j}\right\}\neq \underset{i}{\text{min}}\left\{a_{ik}^{j}\right\}$. Different attributes in MALGDM problems are often measured in different units, so the preference value should decision matrix *V*^*j*^=(*v*_*ik*_^*j*^)_*n*×*P*_ as follows:

(1) $$\begin{array}{c} v_{ik}^{j}=\frac{a_{ik}^{j}-\underset{i}{\min}\left\{a_{ik}^{j}\right\}}{\underset{i}{\max}\left\{a_{ik}^{j}\right\}-\underset{i}{\min}\left\{a_{ik}^{j}\right\}}. \end{array}$$

### 2.2. Clustering Method for Large-Group Members

To simplify the decision-making processes, decision-makers are clustered by individual decision matrix *A*^*j*^ to transform into small-group decision making in LGDM. decision-makers are clustered into *l* clusters (1 < *Y* < *n*) by the improved mean of preference clustering method, which is described in Algorithm 1.

### 2.3. Determination of Consensus Level

Decision-makers' respective weights in the decision processes can be determined by the following definitions:

**Definition 1 .**

Experts in larger subgroups should be given larger weights based on the majority principle [37]. In line with the number and weight values of experts in the subgroup, the subgroup weight value can be defined as

(2) $$\begin{array}{c} \lambda_{l}=\frac{{\left(\sum_{j=1}^{M}\omega_{j}\times \theta_{jl}\right)}^{2}}{\sum_{l=1}^{Y}{\left(\sum_{j=1}^{M}\omega_{j}\times \theta_{jl}\right)}^{2}}, \end{array}$$

where *θ*_*jl*_=1 represents expert *e*_*j*_ belonging to the subgroup *C*^*l*^; *θ*_*jl*_=0 represents expert *e*_*j*_ not belonging to the subgroup *C*^*l*^, where *l*=1,2,…, *L*, and it is easy to know that 0 ≤ *λ*_*l*_ ≤ 1 and  ∑_*l*=1_^*Y*^*λ*_*l*_=1.

**Definition 2 .**

An individual decision matrix can be obtained by decision-makers' opinions, the subgroup decision matrix is computed by aggregating the individual matrices, considering the weights associated with each decision-maker, and the subgroup decision matrix can be calculated [44].

(3) $$\begin{array}{c} r_{ik}^{l}=\overset{M}{\underset{j=1}{\sum }}\omega_{j}\times v_{ik}^{j}\times \theta_{jl}. \end{array}$$

By aggregating single subgroup decision matrixes, the normalized group decision matrix *R*^*c*^=(*r*_*ik*_^*c*^)_*n*×*P*_ is obtained, where *r*_*ik*_^*c*^ is represented as

(4) $$\begin{array}{c} r_{ik}^{c}=\overset{Y}{\underset{l=1}{\sum }}\lambda_{l}\times r_{ik}^{l}. \end{array}$$

**Definition 3 .**

According to the gap subgroup decision matrix *R*^*l*^(*l*=1,2, ···, *Y*) and the group decision matrix *R*^*c*^, the consensus level *CI*(*R*^*l*^) between the subgroups' decision matrices *R*^*l*^(*l*=1,2, ..., *Y*) and the group decision matrix *R*^*c*^ is defined as

(5) $$\begin{array}{c} CI\left(R^{l}\right)=1-d\left(R^{l},R^{c}\right), \end{array}$$

where *d*(*R*^*l*^, *R*^*c*^) is the Manhattan distance between *R*^*l*^ and *R*^*c*^; that is, *d*(*R*^*l*^, *R*^*c*^) represents the similarity between subgroup *C*^*l*^ and *C*^*C*^, which can be defined as

(6) $$\begin{array}{c} d\left(R^{l},R^{c}\right)=\frac{1}{n}\times \overset{n}{\underset{i=1}{\sum }}\overset{P}{\underset{k=1}{\sum }}\eta_{k}\times \left|r_{ik}^{l}-r_{ik}^{c}\right|. \end{array}$$

By calculating the average value of consensus level *CI*(*R*^*l*^), the group consensus level LGCI can be obtained:

(7) $$\begin{array}{c} \text{LGCI}=\lambda_{l}\times \overset{Y}{\underset{l=1}{\sum }}CI\left(R^{l}\right). \end{array}$$

If *LGCI*=1, it indicates that all decision-makers have reached consensus among the large groups. A $\bar{LGCI}$ indicates a higher level of consensus among all decision-makers. $\bar{LGCI}$, as the group consensus threshold, is set to determine whether the consensus reaching process should be carried out. If $LGCI\leq \bar{LGCI}$, the consensus process should be used to change decision-makers' opinions to reach a higher consensus level.

### 2.4. Determination of Consensus Threshold

The consensus threshold $\bar{LGCI}$ should be determined to judge whether the consensus reaching process continues or not. The decision-making pressure of large groups often leads to the uncertainty and subjectivity of the opinion adjustment coefficient. In order to improve the accuracy of group decision making, both objective and subjective factors should be considered. And a coefficient should be set to adjust the subjective factors and objective factors.

#### 2.4.1. Subjective Threshold

For the particular problem, the decision-makers provide the consensus threshold $\bar{LGCI}$ according to the quality of the problem [45]. The consensus threshold $\bar{LGCI}$ reflects its attitude towards the group consensus level and opinion. If the consequence of the decision is important, the consensus threshold should be as high as possible. In this article, let the subjective consensus threshold determined by the decision-makers' experience be ${\bar{LGCI}}_{sub}$. The subjective consensus threshold is the decision-maker's expected consensus level.

#### 2.4.2. Objective Threshold

The objective consensus threshold ${\bar{LGCI}}_{ob}$ is determined to improve the feasibility of the consensus reaching process. The objective consensus threshold ${\bar{LGCI}}_{ob}$ represents that the large group can reach consensus level by the original decision matrices of decision-makers, which is defined as

(8) $$\begin{array}{c} {\bar{\mathrm{LGCI}}}_{ob}=\text{LGCI.} \end{array}$$

**Definition 4 .**

The consensus threshold should satisfy two conditions: one is that the consensus threshold meets the requirement of practical decision problem, which is described as subjective consensus threshold; the other is that the consensus threshold can be reached in the opinion adjustment scale, in which the decision-maker is willing to modify, which is defined as the objective consensus threshold. Thus, it is feasible to determine the consensus threshold by the objective consensus threshold and the subjective consensus threshold:

(9) $$\begin{array}{c} \bar{\mathrm{LGCI}}=\alpha {\bar{\mathrm{LGCI}}}_{ob}+\left(1-\alpha \right){\bar{\mathrm{LGCI}}}_{\text{sub}}, \end{array}$$

where *α* is the consensus threshold adjustment coefficient and defined by the number and weight of decision-makers in the subgroup whose consensus level is less than the group consensus level calculated by the initial group decision matrix.

**Definition 5 .**

Let the subgroup set where consensus level is more than the group consensus level be *G*, which is described as

(10) $$\begin{array}{c} G=\left\{C^{l}|CI\left(R^{l}\right)>\text{LGCI}\right\}. \end{array}$$

The adjustment coefficient *α* can be calculated as

(11) $$\begin{array}{c} \alpha =\frac{\sum_{\lambda =1}^{Y}\lambda_{l}\times \vartheta_{lg}}{\sum_{l=1}^{Y}\lambda_{l}}. \end{array}$$

where *ϑ*_*lg*_ represents the subgroup *l* belonging to *G.*

## 3. Process of Noncooperative Behaviors

In this section, a consensus reaching model suitable for addressing noncooperative behavior in MAGDM problems is proposed. The innovative point of this model features in the abilities to detect and handle individual and subgroup noncooperative behaviors in the consensus reaching process, with the aim of improving the overall consensus reaching process performance.

### 3.1. Noncooperative Behavior Detection

In the consensus reaching model presented in this study, we define an approach to identify those noncooperative subgroups that exist as decision-makers who are reluctant to change their original preferences to reach a consensus, which is aiming at assisting the subsequent treatment of such decision-makers, so as to improve the performance of the consensus reaching process. The detection approach is first used in the second round of the consensus reaching process, because of its requirement of comparisons among subgroups obtained in the previous and current rounds of discussion. There exist three rules to detect the noncooperative subgroup according to the definition of noncooperative behavior. Let the noncooperative subgroup be *C*^*l*^*∗*^^. The detection method includes two steps:

*Step 1*. Determine the detection object
Before the noncooperative subgroup is detected, the detection object should be determined. The noncooperative subgroup includes two common characteristics according to the definition:
(i) The consensus level of the subgroup opinion is smallest, which can be denoted as
  (12) $$\begin{array}{c} C^{l^{*}}=\overset{\underset{Y}{l=1}}{\text{min}}\left\{CI\left(R^{l\left(t-1\right)}\right)|l=1,2,\ldots ,Y\right\}. \end{array}$$
(ii) The number of decision-makers in these subgroups is small, in a general way, which is less than [M/*n*], described by the following formula:
  (13) $$\begin{array}{c} \overset{M}{\underset{j=1}{\sum }}\theta_{jl^{*}}^{t-1}\leq \frac{M}{Y}. \end{array}$$
For example, a large group consists of 15 decision makers, and there are three decision options. The number of people supported by the three decision schemes is 7, 5, and 3, respectively. Their CI value size relation is CI_1_ > CI_2_ > CI_3_. According to characteristic (i), we can know that the third subgroup may be a noncooperative group. We can know that [M/*n*] is 5, the third group's number is 3, and 3 is smaller than 5. So, according to characteristic (ii), the third subgroup can be determined by the detection object.
*Step 2*. Detect the noncooperative subgroup
After the detection object is determined, the subgroup *C*^*l*^*∗*^^ will adjust their decision opinions by discussion in the *t − 1*-th round, and the adjustment decision matrix of decision-maker *A*^*j*(*t*)^ in the subgroup *C*^*l*^*∗*^^ is obtained, and the temporary subgroup decision matrices *R*^'*l*(*t*)^, in which the form of subgroup is not changed, are calculated. For the noncooperative subgroup, there exists at least one decision-maker in the subgroup reluctant to adjust their own opinions, which is checked to determine whether decision-maker in the subgroup *C*^*l*^*∗*^^ is willing to adjust their opinions or not. To do this, the temporary subgroup consensus level *CI*′(*R*^*l*(*t*)^) and the temporary consensus level *LGCI*^′(*t*)^ can be calculated by the temporary subgroup decision matrices *a*^'*l*(*t*)^. Thus, there are three situations to define the noncooperative subgroup:
(i) Decision-makers in *C*^*l*^*∗*^^ are willing to adjust their opinions, but the decision opinion adjustment of decision-maker causes negative influence to the consensus level of *C*^*l*^*∗*^^:
  (14) $$\begin{array}{c} C^{l^{*}}=\left\{C^{l}|CI^{\prime }\left(R^{l\left(t\right)}\right)<CI\left(R^{l\left(t-1\right)}\right);\;l=1,2,\ldots ,Y\right\}. \end{array}$$
(ii) Decision-makers in *C*^*l*^*∗*^^ do not change their opinions, that is, the consensus level of *C*^*l*^*∗*^^ in *t*-th round keeping correspondence with *t-*1-th round:
  (15) $$\begin{array}{c} C^{l^{*}}=\left\{C^{l}|CI^{\prime }\left(R^{l\left(t\right)}\right)=CI\left(R^{l\left(t-1\right)}\right);\;l=1,2,\ldots ,Y\right\}. \end{array}$$
(iii) The decision opinion adjustment of decision-maker causes positive influence to the consensus level of *C*^*l*^*∗*^^, but there are only a small part of decision-makers transforming their opinions, and there exist decision-makers who are not willing to modify their opinions. The noncooperative subgroup detection rule can be expressed as

(16) $$\begin{array}{c} C^{l^{*}}=\left\{C^{l}|CI^{\prime }\left(R^{l\left(t\right)}\right)>CI\left(R^{l\left(t-1\right)}\right);\overset{n_{l}}{\underset{j=1}{\sum }}\sigma_{jl}\neq z_{l,}l=1,2,\ldots ,Y\right\}. \end{array}$$

In the example in step (1), there are three decision-makers in the detection subgroup. If the subgroup has noncooperative behavior, then at least one person did not change his preference, or the decision opinion adjustment of decision-maker causes negative influence to the consensus level of *C*^*l*^*∗*^^. If all decision-makers adjust their opinions, but *CI*′(*R*^*l*(*t*)^) < *CI*(*R*^*l*(*t* − 1)^), this is the first situation of noncooperative subgroup. If all decision-makers do not adjust their opinions, this is second situation. If at least one decision-maker did not adjust his opinion, despite the adjustments made by the rest of the decision-makers, and those adjustments have had a positive impact to the consensus level, subgroups are also considered as noncooperative subgroup. This is the third situation.

where *σ*_*jl*_ represents decision-maker *e*_*j*_ willing to change their opinions, and *σ*_*jl*_ is detected by change degree. The change degree is introduced to measure the decision-maker who modify his opinion [32]; it can be described as

(17) $$\begin{array}{c} \varphi_{jl}^{t}=\frac{1}{n}\overset{n}{\underset{i=1}{\sum }}\overset{P}{\underset{k=1}{\sum }}\eta_{k}\times \left|v_{ik}^{j\left(t\right)}-r_{ik}^{l\left(t-1\right)}\right|\times \theta_{jl}^{t}. \end{array}$$

*σ*_*jl*_ can be computed by comparing the change degree between *t-1*-th round and *t*-th round, which is defined as

(18) $$\begin{array}{c} \sigma_{jl}=\left\{\begin{array}{cc} 1, & \mathrm{if}\;\varphi_{jl}^{t}-\varphi_{jl}^{t-1}\neq 0,\\ 0, & \mathrm{if}\;\varphi_{jl}^{t}-\varphi_{jl}^{t-1}=0. \end{array}\right.. \end{array}$$

### 3.2. Management Strategy of the Noncooperative Behavior

Through Step 3.1, we identified three different types of noncooperative behavior. But the core of this paper is to develop different strategies for different noncooperative behaviors. Thus, unlike the traditional adjustment method for noncooperative behaviors, a new strategy was devised in this study. The management strategy is determined by analyzing the noncooperative degree of noncooperative subgroup. And the noncooperative subgroup is allowed to change. The consensus level needs to be recalculated after the subgroup changed. The concrete process of management strategy is described as follows:

Step 1: measure the noncooperative degree
For the noncooperative subgroup, the degree of noncooperation is used to describe the decision-maker's willingness to change the decision to improve group consensus level. Thus, the degree of noncooperation is influenced by two factors: one is the number of decision-makers who have changed their views, and the other is whether the changed opinion can increase the level of consensus.
The number of policymakers who changed their minds first needs to be counted. In general, the LGCI can reflect whether the noncooperative subgroup modify their initial opinions or not, and the number of decision-makers who are willing to modify their opinions describe the adjustment degree of noncooperative subgroup, which is expressed as
  (19) $$\begin{array}{c} \gamma_{l^{*}}^{t}=\overset{M}{\underset{j=1}{\sum }}\sigma_{jl^{*}}\times \theta_{jl^{*}}. \end{array}$$
And then, Thus, the change of LGCI value can indicate whether a change in decision-makers' opinion has increased the level of group consensus. Thus, the noncooperative degree is measured by the value of *LGCI* and the number of decision-makers who are willing to modify their opinions, which is obtained as follows:
  (20) $$\begin{array}{c} \phi_{l^{*}}^{\left(t\right)}=\frac{\left({\text{LGCI}}^{'t}-{\text{LGDI}}^{t-1}\right)\gamma_{l^{*}}^{t}}{\left|{\text{LGCI}}^{'t}-{\text{LGDI}}^{t-1}\right|z_{l^{*}}}. \end{array}$$
*ϕ*_*l*^*∗*^_^(*t*)^ can represent the degree of noncooperation, and the number of experts in the MALGDM is odd, so *ϕ*_*l*^*∗*^_^(*t*)^ is not equal to 0.5; there are three cases according to the calculation results.
(i) If *ϕ*_*l*^*∗*^_^(*t*)^ ≤ 0, it means that the subgroup *C*^*l*^*∗*^^ manifests a very high degree of noncooperative behavior. A small number of decision-makers in noncooperative subgroup will change their opinions in the *t-1*-th round, and the changed result is negative for the consensus level.
(ii) If 0 < *ϕ*_*l*^*∗*^_^(*t*)^ < 0.5, the subgroup *C*^*l*^*∗*^^ is considered as a partly noncooperative subgroup. A small part of the decision-maker in noncooperative subgroup modified their opinions in the *t-1*-th round, and the change result of subgroup is positive for consensus level.
(iii) If 0.5 < *ϕ*_*l*^*∗*^_^(*t*)^ ≤ 1, it indicates that the subgroup *C*^*l*^*∗*^^ is a cooperative subgroup, and more than half of decision-makers in the subgroup *C*^*l*^*∗*^^ change their opinions, and the change result of subgroup is positive for consensus level.
Step 2: process the noncooperative behavior
When the subgroup's noncooperative degree is got, the novel noncooperative behavior treatment method that considers the change of subgroup is developed. The following strategies are adopted for the three cases with different degrees of noncooperation.
(i) For the noncooperative subgroup, that is, *ϕ*_*l*^*∗*^_^(*t*)^ ≤ 0, the decision-makers in subgroup *C*^*l*^*∗*^^ not only do not improve the group consensus, but also may cause the group consensus decrease. To speed up the decision-making processes and obtain proper results with a short period of time, the subgroup *C*^*l*^*∗*^^ will be suggested to withdraw from the decision process.
(ii) For the partly noncooperative subgroup, that is, 0 < *ϕ*_*l*^*∗*^_^(*t*)^ < 0.5, a major part of decision-makers do not change their opinions in the subgroup *C*^*l*^*∗*^^. It should be penalized by adjusting the weight of subgroup. But there are minor decision-makers willing to modify their decision opinion, and their opinions should be protected. Therefore, we need to determine whether the decision-maker who changes perspective belongs to the subgroup *C*^*l*^*∗*^^. And then the form of subgroup *C*^*l*^*∗*^^ may be changed. Thus, the treatment method is determined by judging whether the decision-makers belong to the subgroup *C*^*l*^*∗*^^.

Let the set of decision-makers who are unwilling to modify his opinion be *C*^'*l*^*∗*^^ in the subgroup *C*^*l*^*∗*^^, and the number is *z*_*l*^*∗*^_′, the set of decision-makers who are willing to modify his opinion is *B*^*l*^*∗*^^. That is, *B*^*l*^*∗*^^={*e*_*j*^*∗*^_*|e*_*j*^*∗*^_ ∈ *C*^*l*^*∗*^^, *e*_*j*^*∗*^_ ∉ *C*^'*l*^*∗*^^}. Based on the (17), we proposed a method to judge whether the decision-maker belongs to the subgroup *C*^*l*^*∗*^^, which is denoted as

(21) $$\begin{array}{c} \varphi_{j^{*}l^{*}}^{'t}=\overset{\underset{z_{l^{*}}^{'}}{q^{*}=1}}{\text{max}}\frac{1}{n}\overset{n}{\underset{i=1}{\sum }}\overset{P}{\underset{k=1}{\sum }}\eta_{k}\times \sigma_{j^{*}l^{*}}^{t}\times \left|v_{ik}^{j^{*}t}-r_{ik}^{q^{*}t}\right|\left(j^{*}\neq q^{*},e_{q^{*}}\in C^{'l^{*}},l^{*}\in Y\right). \end{array}$$

where *φ*_*j*^*∗*^*l*^*∗*^_^'*t*^ represents the conflict degree between *e*_*j*^*∗*^_ and subgroup *C*^*l*^*∗*^^ in the *t*-th round.

  If *φ*_*j*^*∗*^*l*^*∗*^_^'*t*^ ≤ 1 − *ς*, it represents that the decision-maker *e*_*j*^*∗*^_ belongs to the subgroup *C*^*l*^*∗*^^ after his opinion is changed.
  If *φ*_*j*^*∗*^*l*^*∗*^_^'*t*^ > 1 − *ς*, it represents that the decision-maker *e*_*j*^*∗*^_ does not belong to the subgroup *C*^*l*^*∗*^^ after his opinion is changed, and the decision-maker *e*_*j*^*∗*^_ can be clustered based on the procedure in Algorithm 1.

Although there are a little of decision-makers to modify their opinions in partly noncooperative subgroup, the subgroup *C*^*l*^*∗*^^ still expresses the lower cooperative level. Thus, in order to reduce its impact on the group consensus level, the weight of subgroup *C*^*l*^*∗*^^ also needs appropriate adjustment. The modified function is a decreasing function. To describe the interaction between the weight adjustment and the number of decision-makers, the weight adjustment function is developed based on the number of decision-makers who is willing to modify its own opinion, which is defined as

(22) $$\begin{array}{c} \lambda^{\prime }{l^{*}}_{t}=\frac{z_{l^{*}}^{t}-z^{\prime }{l^{*}}_{t}}{z^{\prime }{l^{*}}_{t}}\lambda_{l^{*}}^{t}. \end{array}$$

where *λ*_*l*^*∗*^_^'*t*^ is the weight of subgroup *C*^*l*^*∗*^^ after adjustment in the *t*-th round, and *λ*_*l*^*∗*^_^*t*^ is the weight of subgroup *C*^*l*^*∗*^^ in the *t*-th round. Generally speaking, subgroup weights reflect their contributions to the group consensus level. When the opinions of decision-makers in the subgroup *C*^*l*^*∗*^^ are modified, the subgroup' contributions are adjusted too. The greater the contribution of subgroup to consensus level, the more important it is. Individuals who have changed opinion with negative effect to the group consensus level should reduce some weight [46]. Based on this rule, the contributions of the subgroup are introduced to measure the subgroup weight in the *t*-th round, which can be defined as

(23) $$\begin{array}{c} D_{l^{*}}^{t}={\text{LGCI}}^{t}-{\text{LGCI}}_{\bar{l^{*}}}^{t}. \end{array}$$

where *D*_*l*^*∗*^_^*t*^ represents the contributions of subgroup *C*^*l*^*∗*^^ for the group consensus level. $LGCI_{\bar{l^{*}}}^{t}$ denotes the group consensus level without the subgroup *C*^*l*^*∗*^^ in the *t*-th round, which is defined as

(24) $$\begin{array}{c} {\text{LGCI}}_{\bar{l^{*}}}^{t}=\overset{L}{\underset{l=1,l\neq l^{*}}{\sum }}\lambda_{l}^{t-1}\overset{n}{\underset{i=1}{\sum }}\frac{1}{n}\overset{P}{\underset{k=1}{\sum }}\eta_{k}\times \left|r_{ik}^{l\left(t\right)}-r_{ik}^{c\left(t\right)}\right|. \end{array}$$

To protect the interest of decision-makers who are willing to modify its own opinion, we need to update the weights of the subgroup and recalculate the group-decision consensus level. The following equations show how to update the weights:

(25) $$\begin{array}{c} \lambda_{l^{*}}^{t}=\lambda_{l^{*}}^{t-1}\times {\left(1+D_{l^{*}}^{t}\right)}^{\xi }. \end{array}$$

*ξ* expresses the impact of the subgroups' contributions on their weights, which are usually given by the decision-makers in advance. If the decision problem is in urgency and has to be dealt with in time, it should be assigned less restrictive values. Otherwise, more restrictive values must be put into use.

(iii) For the cooperative subgroup, that is, 0.5 < *ϕ*_*l*^*∗*^_^(*t*)^ < 1, more than half of decision-makers in this subgroup are willing to modify their initial opinions. This subgroup expresses a very high degree of cooperative behavior. Thus, the motivation mechanism should be adopted to protect the decision opinion of this subgroup. Similar to partly noncooperative subgroup, if the decision-maker *e*_*j*^*∗*^_ does not belong to the subgroup *C*^*l*^*∗*^^ after his opinion is changed, Algorithm 1 can be used to cluster the decision-maker *e*_*j*^*∗*^_ into a suitable subgroup. If the decision-maker *e*_*j*^*∗*^_ belongs to the subgroup *C*^*l*^*∗*^^ after his opinion is changed, the motivation mechanism is similar to the treatment process of partly noncooperative subgroup. When the number of decision-makers who modify their own opinions is more than the half of subgroup, the adjustment coefficient of the subgroup in equation (26) is not less than 1. Thus, the weight of subgroup will be enhanced.

### 3.3. Algorithm of Large Group Consensus

Adopting consensus reaching model that the basic thought is to adjust decision information matrix, to enable the decision-makers to have a higher consensus level, the noncooperative subgroup is detected and addressed to obtain a higher consensus level.  Algorithm 2 of the consensus reaching model is summarized as follows.

Let *t*^*∗*^=*t*. Output the final subgroups' decision matrices *R*^*k*(*t*^*∗*^)^(*k*=1,2,…, *n*) and the final group decision matrix *R*^*c*(*t*^*∗*^)^.

**Figure 1:**
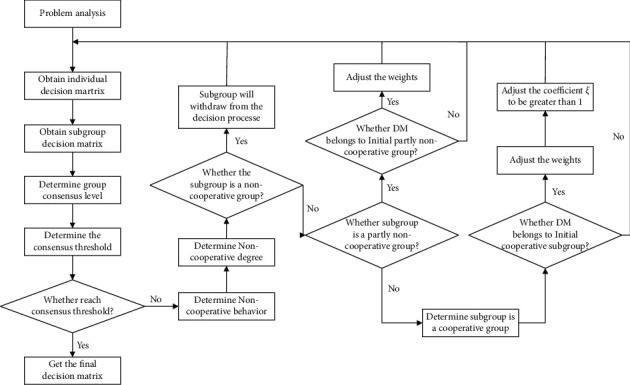
The process of consensus reaching model.

The process of consensus reaching model can be simply described in Figure 1.

## 4. Case Study

In this section, an example of emergency decision-making problem in flood disaster is applied to indicate the feasibility of the presented method.

### 4.1. Case Background

There is a flood disaster hit Hu Nan Province, a south city in China, on July 3, 2018. After the flood disaster, the government carried out an emergency scheme based on instructions. As shown in Table 1, four preliminary plans were rapidly drawn up:

Twenty experts *E*=(*e*_1_,  *e*_2_, ...,  *e*_20_) from different fields were asked to make decisions based on these four alternatives *X*=(*x*_1_, *x*_2_, *x*_3_, *x*_4_). We consider three criteria for each alternative: (1) personnel security rate (*f*_1_), the scale of evaluation value of personnel security rate is 0 to 1; (2) personnel injured rate (*f*_2_), the scale of the evaluation value of the effectiveness of equipment is the same to personnel security rate; (3) the development of situation of flood disaster (*f*_3_).

### 4.2. The Process of Group Decision-Making

We set that the scale of evaluation value of development situation of flood disaster is 1 to 100. Each decision-maker opinion should be seriously taken into account. Suppose that there is no conflict of interest among the decision-makers. In order to obtain the best alternative(s), the following steps need to be performed.

*Step 1.* Cluster the initial normalized individual decision matrices.
To save space, the normalized individual decision matrices are omitted. Base on the clustering method, which is described in Algorithm 1, the clustering threshold is set as *ζ*=0.8, and the group can be divided into several smaller clusters.  Table 2 shows the results, indicating that the original decision group can be divided into five clusters.
*Step 2.* Calculate the group decision matrix.
Aggregate the decision-makers' decision matrices into the subgroups' decision matrices by the (5). The weight of subgroup is calculated by the (2), and the group decision matrix is calculated by the individual decision matrices and the weight of cluster, which is adopted as
  (26) $$\begin{array}{c} R^{c\left(0\right)}=\left[\begin{array}{ccc} 0.5099 & 0.8063 & 0.5721\\ 0.6400 & 0.4345 & 0.3138\\ 0.2837 & 0.7329 & 0.2879\\ 0.4810 & 0.1408 & 0.7346 \end{array}\right]. \end{array}$$
*Step 3.* Consensus measure and calculate the consensus threshold.
Compute the subgroup consensus levels by the (5), that is, *CI*(*R*^1(0)^)=0.7949, *CI*(*R*^2(0)^)=0.7388, *CI*(*R*^3(0)^)=0.6488, *CI*(*R*^4(0)^)=0.7263 and *CI*(*R*^5(0)^)=0.6141. The initial group consensus level can be calculated as *LGCI*(*R*^*c*(0)^)=0.7416. The consensus threshold can be calculated from the subjective and objective aspect, the subjective threshold is set as 0.8, and the objective threshold is 0.7416. The adjustment coefficient is obtained by the (11), which is expressed as 0.2234. Finally, the consensus threshold is computed as 0.7869. Because of *LGCI*(*R*^*c*(0)^)=0.7416 < 0.7869, the consensus process should be applied to change some opinions.
*Step 4.* Consensus reaching process.
(i) First consensus reaching iteration
The detection object of the noncooperative cluster is determined by (12) and (13), and the calculation result shows that the subgroup C^5^ is the detection object. The decision-makers in the subgroup C^5^ modify their initial decision opinions to reach the consensus, the decision-makers who needed to reconsider their preferences were determined, and the modification criterion of the decision-maker is the group decision matrix. The decision matrix of decision-maker *A*^*j*(1)^ can be obtained after modification. The temporary subgroup decision matrix *R*^'5(1)^ can be computed by the decision matrix of decision-maker *A*^*j*(1)^. The consensus level of clusters is calculated by the temporary subgroup decision matrix *R*^'5(1)^, which is expressed as *CI*′(*R*^5(1)^). Comparing the temporary subgroup consensus level *CI*′(*R*^5(1)^) with consensus level *CI*(*R*^5(0)^), the result shows that the *CI*′(*R*^5(1)^) is less than *CI*(*R*^5(0)^), and the subgroup C^5^ is noncooperative subgroup, which denotes that the subgroup C^5^ expressed the high noncooperative behavior.
Based on the noncooperative management method, *ϕ*_*l*^*∗*^_^(*t*)^ ≤ 0, the subgroup C^5^ is suggested to quit the consensus process. Thus, the number of subgroups is null, and the decision matrix is 0, and there are four subgroups by clustering the decision-maker after management. Because of the change of subgroup number, the weight of cluster should be recalculated by (2), which is described as *λ*^(1)^=(0.350.3500, 0.2500, 0.2000, 0.2000, 0). Continuing consensus measure, the new cluster consensus levels are *CI*(*R*^1(1)^)=0.7914, *CI*(*R*^2(1)^)=0.7425, *CI*(*R*^3(1)^)=0.7259 and *CI*(*R*^4(1)^)=0.7238. The group consensus level is *LGCI*(*R*^*c*(1)^)=0.7526 < 0.7869. Thus, the consensus reaching process continues.
(ii) Second consensus reaching iteration
As *CI*(*R*^4(1)^)=min{*CI*(*R*^*i*(1)^)*|i*=1,2,3,4}, the cluster C^4^ can be regarded as detection object in the 2-th round iterations, and the decision-makers in the cluster C^4^ are required to modify their own opinion. The decision-maker's decision opinion in the 2-th rounds can be obtained after discussion, which is described as *A*^*j*(2)^, aggregating the individual decision matrices, obtaining the temporary group decision matrix. The temporary consensus level is determined by the temporary subgroup decision matrix *R*^'4(2)^ and temporary group decision matrix. Comparing the temporary subgroup consensus level *CI*′(*R*^4(2)^) with consensus level *CI*(*R*^4(1)^), the result shows that the *CI*′(*R*^4(2)^) is more than *CI*(*R*^4(1)^), but only part of decision-makers in subgroup C^4^ are willing to modify their opinions; thus, the subgroup C^4^ is noncooperative subgroup.

The conflict degree of subgroup C^4^ is calculated to denote the decision-makers who modify their decision opinion. Based on the result of the calculation, there are four decision-makers in the subgroup C^4^ who are willing to modify their decision opinions. The noncooperative degree of subgroup is computed as 0.5 < *ϕ*_*l*^*∗*^_^(*t*)^ ≤ 1. According to (20), whether the decision-maker who modifies his opinion belongs to the initial subgroup can be judged. The result shows that the decision-maker *e*_5_ does not belong to the subgroup C^4^ after changing their initial decision opinion by the (21), and the decision-makers *e*_15_, *e*_16_ belong to the initial subgroup C^4^. The decision-maker *e*_5_ is clustered by the Algorithm 1; the gathered degree between the decision-maker *e*_5_ and the subgroup C^1^ is 0.9135 > 0.8; thus, the decision-maker *e*_5_ belongs to the subgroup C^1^. The weights of C^4^ and C^1^ are updated according to equation (2) and the number of decision-makers. The new subgroup weight can be denoted as *λ*^(2)^=(0.4517, 0.2438, 0.1976, 0.1069).

The new subgroup consensus levels are *CI*(*R*^1(2)^)=0.8014, *CI*(*R*^2(2)^)=0.7956, *CI*(*R*^1(2)^)=0.7643 and *CI*(*R*^1(2)^)=0.7234. The group consensus level is *LGCI*(*R*^*c*(2)^)=0.7901 > 0.7869. After two iterations, the decision-makers obtain consistency, and the final group consensus level meets the predefined requirement.

Due to the reaching of consensus threshold by group consensus level, the group decision matrix can be used to determine, which alternative is optimal, and the final calculation result of a group decision is expressed as

(27) $$\begin{array}{c} R^{c\left(2\right)}=\left[\begin{array}{ccc} 0.5119 & 0.8313 & 0.5596\\ 0.6358 & 0.4491 & 0.2745\\ 0.2753 & 0.6829 & 0.3379\\ 0.4859 & 0.1408 & 0.7529 \end{array}\right]. \end{array}$$

According to the weight of the attribute, the decision vector is calculated as (0.6343, 0.4531, 0.4321, 0.4599), and the value of alternative *x*_1_ is 0.6343. Thus, the alternative *x*_1_ is the most optimal.

## 5. Discussion

**Figure 2:**
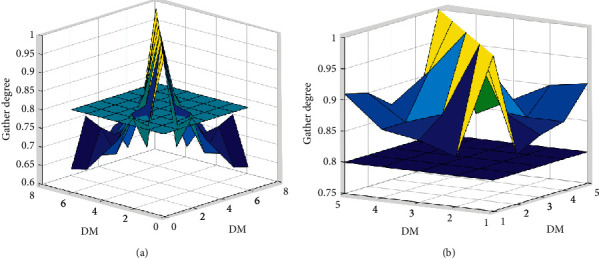
The effect of gather degree of decision-makers of different clustering methods. (a) The gather degree of traditional clustering method. (b) The gathered degree of improved clustering method.

In this paper, we proposed a novel method to calculate the consensus threshold from the subjective and objective aspect. It is unlike the traditional determination method consensus threshold. Based on this consensus threshold and the traditional clustering method, the improved clustering method is developed. The improved method depends on the similarities between the decision-makers' decision opinions and subgroups' decision opinion. To better reflect the advantages of the clustering method, the case in Section 3 is adopted to compare the difference between the current clustering method and the traditional clustering method. We take the subgroup that includes the decision-maker *e*_1_ as example, and the gathered degree of decision-makers for different clustering methods can be computed. And comparing the clustering thresholds, the effect of gather degree is shown in Figure 2.

From Figure 2, we can obtain that there are many decision-makers whose gathered degree is less than clustering threshold for the traditional clustering method (see Figure 2(a)); but it can be seen from Figure 2(b) that the gathered degree for any decision-makers is more than 0.8 after using the improved clustering method. The decision-makers using the improved method have higher gather degree than those using the traditional method. The result shows that the traditional clustering method considers the whole gather degree and omits the gather degree among decision-makers. Thus, the improved clustering method is more suitable to divide into the group.

**Figure 3:**
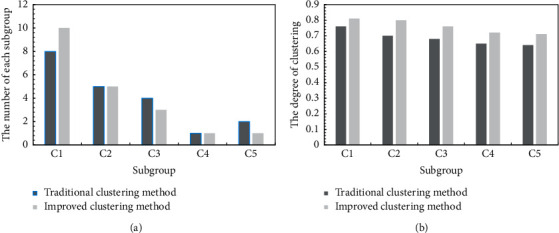
Comparisons between different groups under two different methods. (a) The number of each subgroup under two different methods. (b) The gathered degree of each subgroup under two different methods.

Except for one group comparison, it can also compare the composition of different subgroups and the degree of subgroup clustering. The results of the comparison can be represented in Figure 3.

In Figure 3, groups are more concentrated. And compared to using traditional methods, each group has a higher degree of clustering after using the improved method in this article. The goal of MALGDM is to achieve a high degree of consistency. And the higher degree of clustering represents that the large group has higher consensus level. Thus, the method used in this article is more effective than the traditional one in dealing with MALGDM problem.

Due to adherence to the principle that noncooperative behavior needs full consideration, the group consensus level increased from 0.7416 to 0.7901, which means that the management of the noncooperative behavior is helpful in reaching the consensus level. Generally, the noncooperative behavior can be addressed by two steps: the detection of the noncooperative behavior and the management of the noncooperative behavior. The current detection method uses the subjective adjustment result of subgroup to detect the noncooperative behavior. Unlike the current detection method, the practical adjustment result of subgroup is adopted to judge the noncooperative behaviors, that is, comparing the change degree of the subgroup decision matrix in the *t*-th rounds and *t* − *1*-th rounds.

For the management of the noncooperative behavior, the novel approach is explored to manage the noncooperative behavior. In general, the weight of the subgroup can be adjusted to manage the noncooperative behavior, and the decision matrix is transformed by the adjustment coefficient. In the practical decision problem, the adjustment opinion which the decision-maker is willing to accept should be respected. Thus, the noncooperative behavior is managed by adjusting the weight of subgroup, recalculating the subgroup decision matrix in this paper. In this study, the subgroups are allowed to modify. Generally, the number of subgroups is changed due to an enforced exit rule; nevertheless, the subgroups themselves remained the same. It is assumed that the decision makers can choose to modify their opinions under discussion, which makes it sensible that the subgroups they are classified into may also change. The example in Section 4 validated this point. Meanwhile, the weight of subgroup can be adjusted by the contribution of the cluster in the group consensus level and the number of the decision-makers who are willing to modify their initial opinion in the cluster.

Different decision-making models have different emphasis. Therefore, there is no model that can be referred to as the best. Despite offered valuable methods to handle multiattribute large group decision-making problems, there are limitations in the proposed model: it is not easy to determine the subjective consensus threshold in this paper. Similar to other consensus models, the subjective consensus threshold needs to be decided by the moderator or group. Although empirical values for the consensus threshold can be given, the determination of the subjective consensus threshold depends on the actual problems and simulations. The subjective consensus threshold makes it free for the moderator and/or the group to grasp the decision processes.

## 6. Conclusion

The MALGDM problem becomes more and more significant for participants and stakeholders to make a consensus-based decision. The main contributions of the paper are as follows:

1. A novel clustering method was adopted to divide the large group into several clusters. The similarity is calculated to express the gather degree of decision-makers, and the decision-maker can be classified by the gathered degree. The value of gathered degree decides the number of subgroups.
2. A consensus framework for the consensus reaching process in a MALGDM is proposed. The consensus threshold is determined by the consensus level and subjective consensus threshold. Meanwhile, the noncooperative behavior of decision-makers is determined, and the subgroup that includes the decision-maker who expresses the noncooperative behavior is defined as the noncooperative subgroup.
3. A novel consensus reaching process is designed to address the noncooperative subgroup. The noncooperative subgroup is detected by the number of decision-makers and the consensus level, and by determining three noncooperative behavior situations. The weight of the subgroup is adjusted to reach the consensus level, and the subgroups in our proposed approach are allowed to change. Thus, there are three approaches to manage the noncooperative behavior: adjustment weight of subgroup, quitting the decision process, and changing the subgroups in each interactive round.

Further, some other clustering methods can be incorporated in the proposal to detect the influences of clustering on model convergence. For the classification of large groups, it may be a good alternative to adopt an automatic feedback strategy such as an optimization-based approach. However, some limitations exist in the research. Due to the difference of risk preference, decision-makers may make decisions that are difficult to coordinate, and this paper does not cover the psychological perception of decision makers, which may be an important research direction in LGDM problem in the future. Meanwhile, there are many factors that can influence the decision-making, and the interactions of factors may influence the results. Thus, the approach considering the interactions of factors will be used to explore multiattribute interactions of LGDM.

## Figures and Tables

**Algorithm 1:**
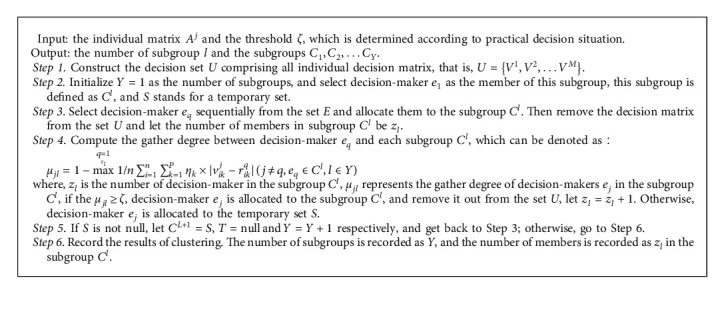


**Algorithm 2:**
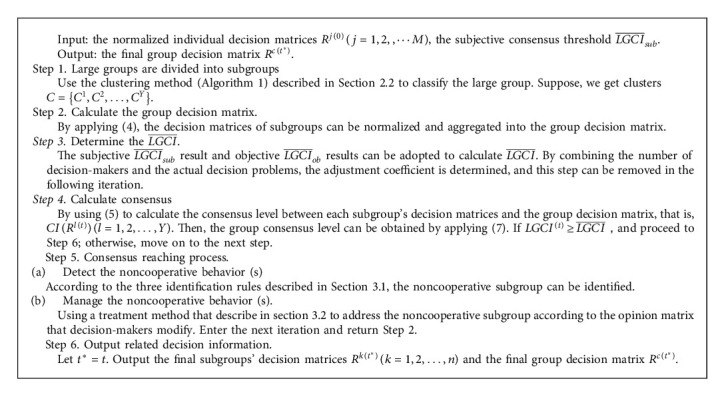


**Table 1:** Different selection strategies.

*x*_*i*_	Concrete measure

*x*_1_	Find out trapped people and evacuate the seriously injured from the disaster areas to avoid further damage caused from flood disaster
*x*_2_	Treat the injured and stop searching for trapped people until the rescue equipment arrived
*x*_3_	Search for trapped people and treat the seriously injured in situ
*x*_4_	Search for trapped people and cease treating the injured until the medical team arrived

**Table 2:** The information of subgroup.

*C* ^*k*^	*n*_*k*_	*e*_*i*_	*λ*_*k*_^(0)^

*C* ^1^	7	*e*_1_, *e*_6_, *e*_7_, *e*_11_, *e*_13_, *e*_17_, *e*_19_	0.3500
*C* ^2^	5	*e*_2_, *e*_3_, *e*_8_, *e*_9_, *e*_18_	0.2500
*C* ^3^	3	*e*_4_, *e*_10_, *e*_20_	0.1500
*C* ^4^	4	*e*_5_, *e*_12_, *e*_15_, *e*_16_	0.2000
*C* ^5^	1	*e*_14_	0.0500

## Data Availability

The data used to support the findings of this study are included within the article.
